# Deep Neural Networks for Automatic Flower Species Localization and Recognition

**DOI:** 10.1155/2022/9359353

**Published:** 2022-04-29

**Authors:** Touqeer Abbas, Abdul Razzaq, Muhammad Azam Zia, Imran Mumtaz, Muhammad Asim Saleem, Wasif Akbar, Muhammad Ahmad Khan, Gulzar Akhtar, Casper Shikali Shivachi

**Affiliations:** ^1^Department of Computer Science, MNS University of Agriculture, Multan, Pakistan; ^2^Department of Computer Science, University of Agriculture, Faisalabad, Pakistan; ^3^School of Information and Software Engineering, University of Electronic Science and Technology of China, China; ^4^Department of Computer Science, Numl, Multan, Pakistan; ^5^Department of Horticulture, MNS University of Agriculture, Multan, Pakistan; ^6^South Eastern Kenya University, Kitui, Kenya

## Abstract

Deep neural networks are efficient methods of recognizing image patterns and have been largely implemented in computer vision applications. Object detection has many applications in computer vision, including face and vehicle detection, video surveillance, and plant leaf detection. An automatic flower identification system over categories is still challenging due to similarities among classes and intraclass variation, so the deep learning model requires more precisely labeled and high-quality data. In this proposed work, an optimized and generalized deep convolutional neural network using Faster-Recurrent Convolutional Neural Network (Faster-RCNN) and Single Short Detector (SSD) is used for detecting, localizing, and classifying flower objects. We prepared 2000 images for various pretrained models, including ResNet 50, ResNet 101, and Inception V2, as well as Mobile Net V2. In this study, 70% of the images were used for training, 25% for validation, and 5% for testing. The experiment demonstrates that the proposed Faster-RCNN model using the transfer learning approach gives an optimum mAP score of 83.3% with 300 and 91.3% with 100 proposals on ten flower classes. In addition, the proposed model could identify, locate, and classify flowers and provide essential details that include flower name, class classification, and multilabeling techniques.

## 1. Introduction 

Flower identification is extremely important in agricultural production, forest management, and other allied sectors. Because of their enormous presence, complex structure, and unpredictable diversity of classes in nature, automated species identification was initially presented 17 years ago [1]. In recent years, the rapid development of technology, flower segmentation, and identification has been an interesting area of research in the image processing and computer vision community. Previous research mostly focused on flower recognition using a conventional detector and technique. Gaston and O'Neill [1] argued that advances in artificial intelligence and digital image processing might generate automated species identification a reality. The rapid development and growing prevalence of key information technologies, along with the widespread availability of compact devices such as digital cameras and smartphones, have resulted in a huge quantity of digital pictures that have been gathered in online databases. As a result, their vision is now almost tangible: mobile devices are utilized to photograph specimens in the field and then identify their species.

With the debut of smartphones and mobile applications, millions of plant pictures have been acquired [2]. Real-world social-based ecological surveillance [3], invasive exotic plant monitoring [4], environmental science popularization, and other applications rely on mobile-based automated plant identification. Scholars and engineers are paying more attention to improving the effectiveness of mobile-based plant identification models [5]. On an extensive dataset, the researchers used a combination of characteristics to enhance flower classification efficiency. The local form/texture, the shape of the border, the overall geographical distribution of petals, and the color are all defined by distinct characteristics. A multiple support vector machine (SVM) classifier was utilized for classification. Fernando [6] developed an image classification-based approach for differentiating feature fusion, in which the color and shape characteristics are merged by the logistic regression strategy for flower picture classification. However, developing an automated flower category classification system remains a difficult challenge due to certain similarities within classes. The textural characteristics from the Gabor replies and the intensity co-occurrence matrix were utilized to automatically classify flowers using the K-nearest neighbor (KNN) classifier [7]. Color texture moments, gray-level co-occurrence matrix, and Gabor responses were used to classify flower pictures [8]. A classifier was built using a probabilistic neural network. The author [9] used a neural network for logistic regression on flower picture characteristics to solve the problem of flower classification. An aspect-based method for flower identification has been proposed in [10]. Visual characteristics were retrieved and generalized to fresh photographs of unidentified flowers to characterize flower pictures. A sparse representation classifier forecasted the characteristics of a particular flower picture. Several techniques depend on human participation [11]. Following years of research and development, smartphone applications like LeafSnap [12], Pl@ntNet [2], and Microsoft Garage's Flower Recognition app [13] are used to identify flowers rapidly.

The technique of flower identification is an essential component of conventional plant ecology research processes. In this research work, photos of various flowers using mobile devices or digital cameras subsequently have been acquired. The name and other information about the blooms from the horticultural expert of MNS-UAM were found uot. It would be really useful to accelerate this work and make it more accessible to nonexperts. Manual flower species identification may be difficult to scale to high-throughput needs even for specialists although it may be prohibitively time-consuming and erroneous for nonexperts. Furthermore, existing models only classify images, limiting the model's ability to recognize numerous flowers in a single image. In contrast, our proposed framework classifies with localization, allowing it to recognize countless flowers in a digital image by placing a boundary box around the identified flower with the label.

## 2. Proposed Methods

We used an eighth Generation Core i7 Quad-Core Processor with 8 GB RAM, 8 GB NVIDIA, and a 520 GB SSD hard drive for our research. This research is divided into four different sections. The first section discusses dataset collection. Image labeling is described in the second section. The third section contains the training of the model on different hyperparameters. The fourth and final section illustrates the model's testing and evaluation with varying inputs from different angles.

### 2.1. Implementation Detail

Some key hyperparameters are introduced to the proposed network, such as the anchor initialization, the maximum number of bounding boxes retained, and the learning rate decay. We initialize our anchors on each pixel of the feature map obtained from the base net. This equates to placing anchors every 16 pixels on each screen dimension. An initialized total of nine anchors will be created for each pixel of the feature map. Three different scales and three different height-width ratios are used to create the nine anchors in the image. 0.5, 1.0, and 2.0 are the scales we use. It has 0.5, 1.0, and 2.0 dimensions. We also need to use the target bounding boxes as labels to train our RPN. Anchors that overlap with the ground-truth box more than RPN POSITIVE OVERLAP = 0.60 are chosen as foreground labels. Anchors that overlap with any ground-truth box less than RPN NEGATIVE OVERLAP = 0.40 are chosen as negative or background labels.  Table 1 shows the training hyperparameters for four nets we trained, inception V2, ResNet50, ResNet101, and MobileNet V2 SSD.

### 2.2. Image Acquisition

This research adopted specific techniques to collect datasets inspired by Michael Rzane and Zhenzhen Song's papers [1, 2]. An image-capturing scheme was developed to collect data on different classes of flowers. The dataset was collected by ourselves, and therefore no web scraping tool was used. A Canon EOS 2000D DSLR was employed to collect data and yield high-resolution images with 2976 × 1984 dimensions with a bit depth of 24. The ordinary lens of the Canon EOS 2000D DSLR with “IntelliAuto mode” was used to capture images. The color representation of each image is sRGB with 72dpi horizontal and vertical resolution. Out of 10 classes, seven classes were obtained from the university garden and 3 classes from the local park. The images were captured from different angles and lens focus (Zoom In, Out).

For an entirely distinct dataset, images of each class were taken at different periods (morning, afternoon, and evening) with different lighting conditions, direct sunlight, shadows, and flashlight in the evening. The images were saved in JPEG format. Each class comprises three images of the same flower from predefined perspectives (entire flower, frontal, and lateral view); (1) the whole flower: it is an image of the whole flower from its natural position on the plant; (2) although the side view of the flower in this group will be comparable to the entire flower image, this study primarily focused on the side view of the flower in this group; (3) flower top view: for this view, we used focus mode instead of “IntelliAuto mode” to focus on all the flower's leaves. Most of the images are of this type in the dataset.

**Figure 1:**
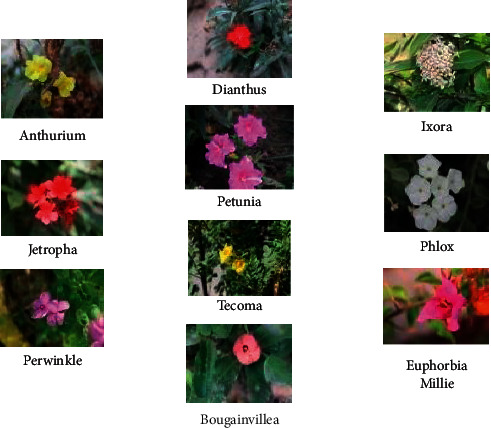
Categories of flowers.

The ten summer season classes are selected for the dataset category containing ten species of flowers as shown in Figure 1 such as Petunia, Dianthus, Jatropha, Periwinkle, Europhobia Milli Phlox, Ixora, Tacoma, Anthurium, Bounganwellia.

### 2.3. Data Labeling

**Figure 2:**
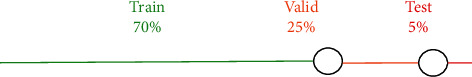
Dataset splitting ratio.

In this study, 2000 pictures are captured. There are up to 15 target flowers objects in each picture, with a resolution of 2976 × 1984 pixels (ROI). To identify pictures with localization, we categorized the flower dataset with annotation comprising ten types of flowers, each with roughly 200 images. When we label the 2000 images, we get 3500 total objects. To acquire the ROI (Region of Interest), we used the GitHub application tools named tagging and marked the area to be selected and then identified photos one by one to offer information about the images. The data labeling is saved in an XML file once the image has been tagged. The XML file includes variables (*w* = width; *h* = height) that form a rectangle in the picture. Since these flowers are usually in the shape of a group, we labeled this composite flower a single flower head (i.e., Ixora). Each class's dataset is split into three folders at random: training, validation, and testing, as shown in Figure 2.

### 2.4. Data Augmentation

Data augmentation is a technique used to increase data size by adding slightly modified copies of existing data or creating new synthetic data from existing data. This technique is used to reduce overfitting when training machine learning models.

We have used adding noise, cropping, flipping, scaling, brightness, and rotation for data augmentation. In adding noise, we added some noise like a blur for viewing the data more accurately. In cropping, we select some parts, crop the image, and resize the original image size. While flipping, the images are flipped horizontally and vertically. While scaling, we scaled the images outward and inward; this way, an image can be more minor and more significant by its original size. Brightness is a process in which we can change an image into brightness and darkness. This technique allows the model to view an image as brightness and lighter. In rotation, the idea is to rotate by a degree ranging from 0 to 360 degrees from its original position. Every rotated image will have a unique representation in the model. When we complete the data augmentation, we have 6540 whole ideas and obtain 10080 natural objects.

### 2.5. Construction of SSD MobileNetV2

The SSD (Single Shot MultiBox Detector) is a fast flower identification detection model based on a single deep neural network [14]. An SSD could simultaneously eradicate multiple target detection and forecast targeted segments and binding boxes.

**Figure 3:**
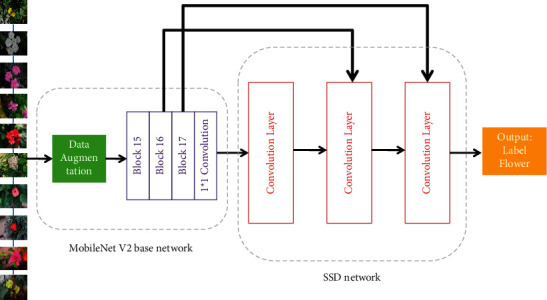
SSD mobile net V2 framework.

The feedforward convolutional network is being used in the SSD model. Its backbone is VGG16, and it follows the primary network with six layers of different characteristics. The size of the inserted map is decreased layer by layer, employing six distinct feature layers to achieve a target of varied scales: low predictive levels and high predictive levels. To forecast the bounding box set of various sizes, the range of objects, and associated confidence, a substantial majority of multiple-choice selections are performed on distinct levels of information. To address the issue of excessive parameter size and training model efficiency, the convolution layer on the actual SSD is substituted by a mobile net splitting layer, which enhances the efficiency and performance of real-time flower recognition as shown in Figure 3.

Real-time object accessibility is also available in the app store, thanks to the MobileNetV2 developers. SSDLite, a hybrid of SSD Object Detector and MobileNetV2, was introduced. Remember how, in the CNN Model, we employed ssd_mobilenetv2 to identify a flower object in images? SSDLite is just the same way. The purpose of using an SSD is straightforward. Since the SSD lacks a whole network, conferences are substituted by a highly fragmented convolution. For MobileNetV2, the very first layer of SSDLite is attached to the extension of layer 15. The set of parameters required by the network to detect an item is substantially reduced when the usual combination is supplanted by intensely split convolution.

When we apply the construction of SSD MobileNetV2, first we apply Data Augmentation, which means the images will be trained in a different dimension; after that convolution filter will be used and the output image is obtained.

### 2.6. Construction of Faster R-CNN Inception V2

**Figure 4:**
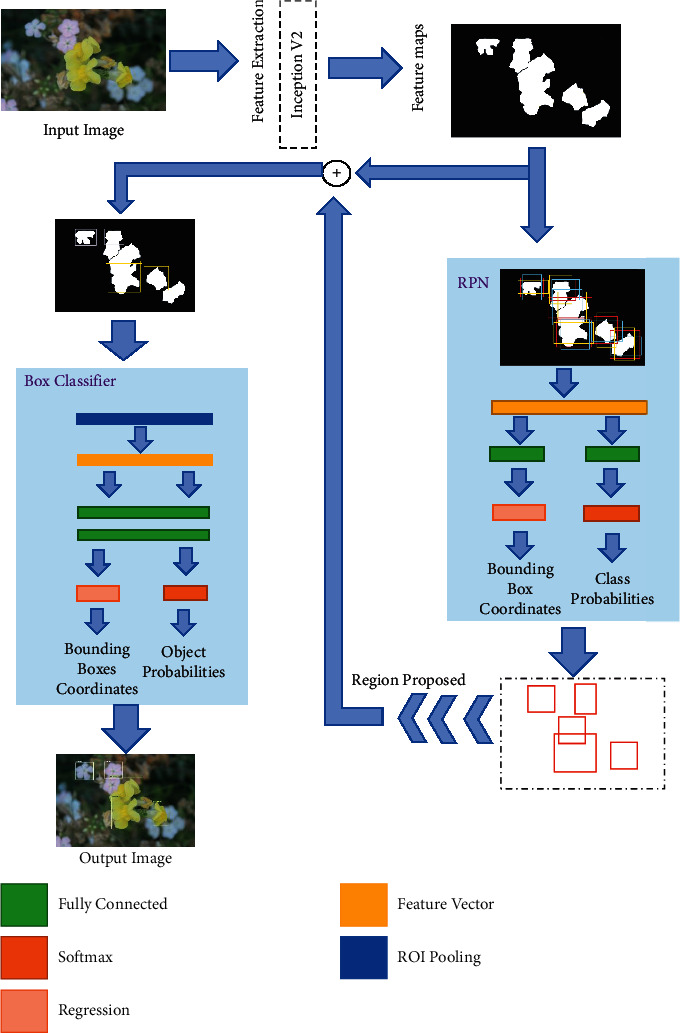
Faster R-CNN flower identification using Inception V2 architecture for feature extractor.

Faster region-based convolution neural network architecture is shown in Figure 4. This architecture was proposed by [15], and it uses Inception V2 for feature extraction, as explained in [16]. Each image was parted into subregions during the preprocessing of the model, as shown in Figure 4, “image division with overlay.”

After that, inception V2 [15] generates the convolutional map feature that has been used in two stages Inception V2 [15] and Faster-RCNN [16]. In Regional Proposed Network (RPN), a convolutional network is used at the first stage that relays over the feature map, extracted by Inception V2, while anchors are placed on each point. Using two similar fully connected layers, the coordinates of the rectangular box around the flower and its probability of accuracy to matching class are determined. In the next step, the determined regions of the image are used to draw a feature map and classify each and every ROI (Region of Interest) with localization. After that, all extracted ROIs are passed through the pooling layer and fully connected layer to determine their probability of classification and localization [17]. These ROIs define the flower's location with a matching class in the output image, as shown in Figure 4.

At last, all output images are transposed and joined to show the original image as they are “divided with overlay” in Figure 1 and they came from training or testing datasets. The bounding boxes were refined and decreased in number by using the suppression algorithm [18]. All this was done by using Tensor Flow and anaconda prompt. Final selected hyperparameters for the next model Faster-RCNN are shown in Table 1 after refining. We give input image to the model Inception V2 applied on it, and feature extraction has been applied. RPN (Region Proposal Network) has been implemented using fully connected layers, activation function, regression feature vector, and ROI pooling. After the proposed region is located in the image, it converts into a Box Classifier, in which two functions are performed: (1) Bounding Box Coordinates and (2) Object Probabilities. The output shows the classification of the input image with a boundary box around the flower.

The output shows the classification of an input image with a boundary box around the flower.

#### 2.6.1. Inception V2

**Figure 5:**
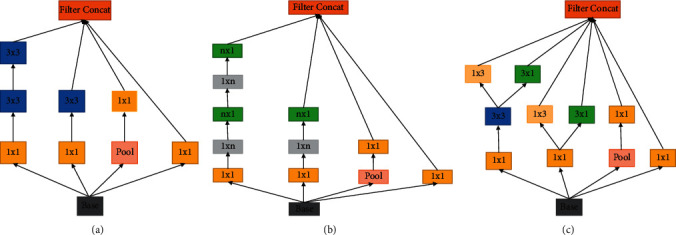
Inception V2 modules A, B, and C using 3 × 3 convolution.

The Inception V2 package was created to decrease the intricacy of the convolution network in flower identification. This control system expands the convolution network rather than making it deeper. Functionalities in Inception V2 are classified into three modules: A, B, and C. It substituted a 3 × 3 convolution for the 5 × 5 convolution. This adheres to the idea that spatial aggregation may be performed over lower-dimensional embedding with little or no loss of representational capacity. Convolution performance was improved by using the 3 × 3 convolution. They discovered that dividing convolution filter size *n* × *n* into 1 × *n* and *n* × 1 convolutions made their technique 33% cheaper than a single 3 × 3 convolution. Additionally, the filter was enhanced to adhere to the concept that higher-dimensional depictions are simpler to process natively inside a network as shown in Figure 5.

#### 2.6.2. ResNet 50

There are 48 Convolution layers in ResNet50 [19, 20], 1 MaxPool layer, and an Average Pool layer. The layers fitted a residual mapping and labeled it as *H*(*x*), and the nonlinear layers fitted another mapping *F*(*x*) = *H*(*x*) − *x*, so the original mapping became *F*(*x*) + *x*. The total number of floating-point operations is 3.8 × 10*∗*9.

#### 2.6.3. ResNet 101

Residual connections can be divided into two categories:

(1)   Identity shortcuts (*x*) may be directly used for inputs and outputs with the exact dimensions.
  (1) $$\begin{array}{c} y=f\left(x,\left\{W_{i}\right\}\right)+X. \end{array}$$
Residual block function is with the same input and output dimensions as Equation (1).
(2) When the dimensions change, (A) identifiability mapping is still performed, with extra zero entries padded with the increased dimension. (B) Using a projection shortcut, the dimension can be matched (for example, by using 1*∗*1 Conv) using the following formula:

(2) $$\begin{array}{c} y=f\left(x,\left\{W_{i}\right\}\right)+W_{s}X. \end{array}$$

Equation (2) deals with residual block function, which has different input and output dimensions.

## 3. Experiment Results and Performance Analysis

A series of comparisons of different object detection models have been conducted to evaluate the effectiveness of the proposed integrated approach. In this research, three object detection models are implemented. During the experiment, we have (1) trained the SSD and Faster-RCNN over ten flower clas images and analyzed its performance and (2) trained the object detection models using a transfer learning approach on different backbones that include Inception V2, ResNet 50, ResNet 101, and Mobile Net V2.

### 3.1. Quantitative Analysis of Flower Detection Performance

Furthermore, the performance of both approaches using qualitative and quantitative measurement methods has been compared. Several evaluation metrics were used to measure the effectiveness of flower detection for quantitative analysis, including the Mean Average Precision (mAP), Average Recall (AR), and average precision (AP). It is most commonly used to calculate the Precision and Recall of measurement systems based on the following equations:

(3) $$\begin{array}{c} \text{Precision}=\frac{\text{TP}}{\text{TP}+\text{FP}}, \end{array}$$

(4) $$\begin{array}{c} \text{Recall}=\frac{\text{TP}}{\text{TP}+\text{FN}}, \end{array}$$

(5) $$\begin{array}{c} F1=\frac{2*\text{Precision}*\text{Recall}}{\text{Precision}+\text{Recall}}. \end{array}$$

TP stands for true positives, FP for false positives, and FN for false negatives. Further, the correctness of a positive is assessed through FPs and FNs evaluated by the intersection over union (IoU) overlap with the corresponding ground-truth bounding box [21]. It is calculated according to the following equation:

(6) $$\begin{array}{c} \text{IoU}=\text{Area}\frac{\text{of}\cap }{\text{Areaof}\cup \;}. \end{array}$$

Detected objects that were not matched to the ground-truth bounding box were considered false positives (FP). If the IoU (6) exceeded the threshold, it was considered a true positive (TP). Furthermore, a false negative (FN) is identified in the missed ground-truth bounding box. We have chosen 0.5 and 0.75 as the threshold values for this study. Detection performance has been determined by averaging the mean average precision (mAP) score and AP values from all classes [22]. As mAP increased, the overall performance of the flower dataset improved. Tables 2 and 3 show that these different object detection models have different detection performance results [20].

A summary of the Precision (given equation (3)) and Recall (given equation (4)) of different detection models is presented in Tables 1 and 2. With the different AP IoU (0.5:0.95, 0.50, and 0.75) 0.81, 0.92, and 0.91, the proposed model Faster R CNN inception V2 with 100 proposals gives the best performances. Moreover, the values of different AR detections (1, 10, and 100) were 0.77, 0.91, and 0.89, respectively. We calculate *F*1-score using mean precision @0.5 IoU and recall @10 concerning 100 and 300 proposals, as shown in Table 4.

### 3.2. Qualitative Analysis for Different Flower Detection Experiment Results

**Figure 6:**
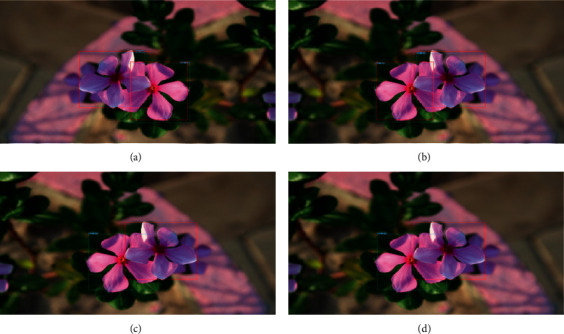
Performance of object detection models for Faster-RCNN using (a) Inception, (b) ResNet50, (c) ResNet101, and (d) SSD using MobileNet V2.

The intersection qualitatively evaluates the correctness of a detected object over union (IoU) overlap with the corresponding ground-truth bounding box [14]. The ground-truth bounding boxes are those hand-labeled boxes from the training set indicating where the flowers are on the image. An example of the IoU overlap is seen in Figure 6, and this suggests that the prediction bounding box will be evaluated using IoU (i.e., prediction of object detection model).

**Figure 7:**
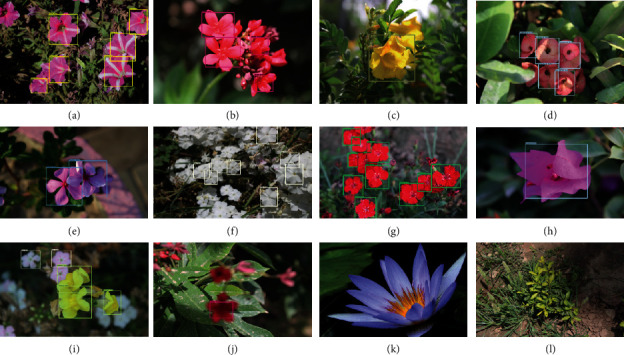
Output of testing images of different classes: (a) Petunia; (b) Jatropha; (c) Tacoma; (d) Europhobia milli; (e) Periwinkle; (f) Phlox; (g) Diahtus; (h) Bouganwelia; (i) Anthrium; (j) Jatropha; (k-l) Nulls.

Across all object detection models, the flower dataset has shown good performance. The red box indicates the ground-truth label, and the boxes of colored lines indicate prediction bounding boxes [23]. We compared the object detection model results to examine the effect of different pretrained CNN architectures on flower detection performance [24]. Figure 6 shows some quantitative results for flower classes.  Figure 7 shows the performance of Faster-RCNN. Figures 7(a)to 7(j) are part of the dataset, and Figures 7(k) and 7(l) are not part of the data used to determine the model generalization.

## 4. Conclusion

An efficient and generalized deep convolution neural network (DCNN)-based model for flower detection, localization, and classification has been proposed. The proposed model localization and recognition of flower species provide flower names, class classification, and multilabeling techniques. This study demonstrated that some classes are very similar in shape and color. In contrast, others can be distinguished better by their external shapes than their internal shapes and vice versa. Faster-RCNN and other object detection models have been evaluated using pretrained models of the COCO dataset. The proposed model provides an optimum mAP score of 83.3% with 300 and 91.3% with 100 proposals on the flower class dataset up to 100% accuracy confidence. However, it still has some limitations due to color similarity between the two classes of flowers, as shown in Figures 7(c) and 7(i). Furthermore, a multilabel classification model provides botanical information about a flower to help farmers, horticulture, and nonbotanists understand what type of flower it is. We should jointly train the models with visually similar classes as a future step.

## Figures and Tables

**Table 1:** Selected hyperparameters.

Net	Optimizer	Decay epoch	Total epoch	Bach size	GPU
Inception V2	Momentum	8	30k	4	2
ResNet50	SGD	8	50k	4	2
ResNet101	SGD	8	55k	4	2
MobileNet V2 SSD	Momentum	8	200k	4	2

**Table 2:** The performance of Average Precision (AP) for different object detection models.

Object detection model	Backbone pretrained model	AP, IoU					
		@ [IoU = 0.5 : 0.95]		@ [IoU = 0.5]		@ [IoU = 0.75]	
		@ 100 proposals	@ 300 proposals	@ 100 proposals	@ 300 proposals	@ 100 proposals	@ 300 proposals
Faster-RCNN	Inception V2	0.71	0.65	0.91	0.83	0.81	0.74
	ResNet 50	0.69	0.61	0.76	0.79	0.73	0.66
	ResNet 101	0.75	0.68	0.86	0.77	0.79	0.71

SSD	MobileNet V2	0.65		0.76		0.69	

**Table 3:** The performance of Average Recall (AR) for different object detection models.

Object detection model	Backbone pretrained model	AR, detections					
		@1		AR@10		AR@100	
		@ 100 proposals	@ 300 proposals	@ 100 proposals	@ 300 proposals	@ 100 proposals	@ 300 proposals
Faster-RCNN	Inception V2	0.81	0.77	0.83	0.79	0.84	0.80
	ResNet 50	0.8	0.71	0.81	0.76	0.84	0.78
	ResNet 101	0.72	0.58	0.74	0.59	0.76	0.61

SSD	MobileNet V2	0.65		0.66		0.67	

**Table 4:** The performance of F1-score for different object detection models.

Object detection model	Backbone pretrained model	F1-score	
		@ 100 proposals	@ 300 proposals
Faster-RCNN	Inception V2	0.87	0.81
	ResNet 50	0.78	0.77
	ResNet 101	0.79	0.66

SSD	MobileNet V2	0.71	

## Data Availability

The data used to support the findings of this study are available from the corresponding author upon request.
